# Mitochondrial Dynamics and Bioenergetic Alteration During Inflammatory Activation of Astrocytes

**DOI:** 10.3389/fnagi.2020.614410

**Published:** 2020-12-10

**Authors:** Md Habibur Rahman, Kyoungho Suk

**Affiliations:** ^1^Department of Pharmacology, School of Medicine, Kyungpook National University, Daegu, South Korea; ^2^BK21 Plus KNU Biomedical Convergence Program, Department of Biomedical Sciences, School of Medicine, Kyungpook National University, Daegu, South Korea; ^3^Brain Science and Engineering Institute, Kyungpook National University, Daegu, South Korea

**Keywords:** glia, astrocyte, mitochondria, fission, fusion, metabolism, neuroinflammation

## Abstract

Mitochondria are essential cellular organelles that act as metabolic centers and signaling platforms and have been identified as an important subcellular target in a broad range of neuropathologies. Studies on the role of mitochondria in neurological disorders have primarily focused on neurons. However, dysfunctional mitochondria in glial cells, particularly astrocytes, have recently gained research attention due to their close involvement in neuroinflammation and metabolic and neurodegenerative disorders. Furthermore, alterations in mitochondrial energy metabolism in astrocytes have been reported to modulate cellular morphology and activity and induce the release of diverse proinflammatory mediators. Moreover, emerging evidence suggests that dysregulation of mitochondrial dynamics characterized by aberrant fission and fusion events in glial cells is closely associated with the inflammatory activation of glia. In this mini-review, we cover the recent advances in the molecular aspects of astrocytic mitochondrial dynamics and their metabolic changes under the pathological conditions of the central nervous system (CNS).

## Introduction

Astrocytes are homeostatic cells of the central nervous system (CNS), comprising around 40% of the glial population (Pelvig et al., 2008), and exhibit remarkable morphological and functional heterogeneity. Besides providing metabolic and physical support to neurons, astrocytes regulate the blood-brain barrier (Posada-Duque et al., 2014), the extracellular balance of ions and neurotransmitters (Sofroniew and Vinters, 2010), synaptogenesis (Baldwin and Eroglu, 2017), and axon pathfinding, thus rendering these glial cells necessary for brain homeostasis. Studies have demonstrated that astrocytes receive and carry information to other neural cells in a coordinated effort in response to diverse CNS insults, including injury and infection, and initiate reparative mechanisms through the activation of immune defenses (Sofroniew, 2009; Bylicky et al., 2018).

Astrocyte-mediated neuroinflammation is associated with metabolic and degenerative pathologies such as Alzheimer’s disease (AD; Fu and Jhamandas, 2014), Parkinson’s disease (PD; Cabezas et al., 2014), multiple sclerosis (Brosnan and Raine, 2013), traumatic brain injury (Barreto et al., 2011), diabetes or obesity (Rahman et al., 2018), and aging (Zhang et al., 2017). In response to CNS insults, such as injury and diseases, astrocytes undergo a morphological, biochemical, transcriptional, and functional transformation termed as astrogliosis or astrocyte reactivity, which leads to a spectrum of heterogeneous changes in a context-specific manner that vary depending on the etiology and severity of the CNS insults (Sofroniew, 2009; Anderson et al., 2014; Escartin et al., 2019). This inflammatory phenotypic change of astrocytes is characterized by higher expressions of glial fibrillary acidic protein (GFAP), aldehyde dehydrogenase one family member L1, and vimentin proteins, accompanied by their hypertrophic morphology and thick processes (Pekny et al., 1999; Pekny and Nilsson, 2005). Consistent with this, Escartin et al. (2019) have extensively discussed the common features and the core hallmarks of reactive astrocytes in their recent review and suggested that GFAP overexpression and morphological changes are the most common marker for reactive astrocytes. During this process, astrocytes regulate their metabolism to generate lactate, glutamate, and ketone bodies as energy substrates for neurons, as these nutrients are deprived in the lesioned brain (Auestad et al., 1991; Dienel, 2013). However, under specific circumstances, astrogliosis exerts harmful effects such as excessive inflammation through diverse secretory mediators and interference with axon growth and synapse sprouting (Sofroniew and Vinters, 2010).

In recent years, there has been an increasing research focus on glial mitochondrial dynamics and energy metabolism, particularly in the context of the phenotypic transitions of microglia and astrocytes (Motori et al., 2013; Nair et al., 2019). It has been reported that alterations in mitochondrial energy metabolism in these glial cells modulate the cellular morphology and activity. In agreement with this, the phenotypic changes in glia are driven by a mitochondrial metabolic shift; in particular, switching from oxidative phosphorylation to glycolysis is associated with the inflammatory activation of glia in diverse neuroinflammatory conditions (Jiang and Cadenas, 2014; Nair et al., 2019). A previous study reported that increased glycolysis fuels the energy-intensive processes required for the inflammatory activities of immune cells (Ganeshan and Chawla, 2014). Furthermore, alterations in mitochondrial morphology in glial cells, characterized by aberrant fission and fusion events, were found to be closely associated with neuroinflammation (Motori et al., 2013; Kim et al., 2019). In this mini-review, we summarize the recent advances in the molecular aspects of mitochondrial dynamics and bioenergetics in inflammatory astrocytes in the context of metabolic and neurodegenerative diseases.

## Pathways Linking Mitochondrial Dysfunction to Inflammatory Activation of Astrocytes

In diverse neuropathologies associated with excessive and acute/chronic neuroinflammation, including neurodegenerative diseases, traumatic brain injury, and stroke, astrocytes exhibit a highly reactive state (Sofroniew, 2009) and release a variety of pro-inflammatory and anti-inflammatory mediators, which have been implied to contribute to worsening or ameliorating the brain pathology (Bush et al., 1999; Okada et al., 2006). In recent years, multiple pathways, involving morphological and functional routes, have been identified that link mitochondrial dysfunction and inflammatory activation of astrocytes.

### Altered Mitochondrial Dynamics During Inflammatory Activation of Astrocytes

The mitochondrion of mammalian cells is tubular and is constantly maintained by fusion and fission reactions and through the exchange of different genes and proteins; these processes are essential for its appropriate functioning (Chang and Reynolds, 2006). These processes involve a group of proteins, such as dynamin-related protein (DRP1) and fission 1 protein mediating fission event, mitofusin 1 and 2 (MFN1 and 2), and optic atrophy 1 (OPA1) mediating fusion event (Liesa et al., 2009). An appropriate balance between fusion and fission reactions is essential for maintaining the mitochondrial architecture and distribution of mitochondrial components (Liesa et al., 2009). Breakdown of mitochondrial dynamics leads to mitochondrial damage, a condition that has been suggested to be associated with neuroinflammation, aging, and metabolic and neurodegenerative diseases (Detmer and Chan, 2007; Joshi et al., 2019; Liu et al., 2020).

Astrocytic mitochondria play a vital role in brain energy metabolism and immune modulation (Belanger et al., 2011; Joshi et al., 2019). Accumulating evidence demonstrates that the alteration in the metabolic signature of astrocytes reflects their response to neuroinflammation. In CNS pathologies associated with neuroinflammation, astrocytes alter their phenotypes into a reactive state, which results in mitochondrial alterations. A study conducted by Motori et al. (2013) provided insights into the astrocytic mitochondrial changes that occur in injured brain tissues. Upon inflammatory insults, astrocytes exhibit multiple forms of reactivity in different areas of the lesioned mouse brain, which affect the mitochondria in particular. The balance between mitochondrial fusion and fission events is altered in astrocytes during neuroinflammation and tissue injury, and this faulty regulation of the mitochondrial dynamics has been associated with astrogliosis. A highly damaged and proinflammatory brain environment leads to excessive fission of mitochondria in astrocytes, accompanied by an increase in the expression of phosphorylated-DRP1 (Ser^616^), which also induces mitochondrial fragmentation. This phenomenon has been correlated with impaired mitophagy, a cargo-specific subset of autophagy (Sheng, 2014). It has been suggested that these alterations in astrocytic mitochondrial dynamics play a critical role in cellular aging and trigger neuroinflammation and neurodegenerative diseases. A previous study identified a correlation between the alterations in mitochondrial dynamics in astrocytes and the neuroinflammation in mice with chronic manganese-induced neurotoxicity (Sarkar et al., 2018). That study demonstrated that manganese treatment increased mitochondrial circularity and decreased MFN2 levels inducing an excessive mitochondrial fragmentation along with inflammatory activation of astrocytes characterized by an elevated GFAP expression. However, mito-apocynin, a mitochondria-targeted antioxidant, significantly attenuated the manganese-induced expression of inflammatory genes and astrogliosis.

Increased mitochondrial fission and fragmentation have been implicated in neuroinflammation and pathogenesis of degenerative diseases. The role of mitochondrial fragmentation during the inflammatory activation of astrocytes has been reported by Joshi and group in the context of neurodegenerative diseases (Joshi et al., 2019). They demonstrated that excessive fragmented and damaged mitochondria released from microglia cause inflammatory activation of astrocytes, which has been suggested to potentiate inflammatory neurodegeneration in mouse models of AD, Huntington’s disease (HD), and amyotrophic lateral sclerosis. In this study, the mitochondrial fragmentation in microglia was mediated by DRP1–FIS1 (a mitochondrial fission 1 receptor) pathway. While the extracellular damaged mitochondria were detrimental, a transfer of functional mitochondria was neuroprotective. The amounts of extracellular functional mitochondria were inversely proportional to the number of damaged mitochondria under pathological conditions. Also, the mitochondrial dysfunction in glial cells has been characterized by lower ATP levels, loss of mitochondrial inner membrane polarization, and increased mitochondrial reactive oxygen species (ROS) production, which has been correlated with astrocyte activation toward the A1 proinflammatory state. Like microglia, reactive astrocytes also display dysfunctional mitochondria and induce neuronal damage (Joshi et al., 2019). However, a selective heptapeptide inhibitor (P110) of excessive mitochondrial fission and fragmentation was found to suppress glial activation, neuroinflammation, and neurodegenerative phenotypes *via* inhibiting the binding of activated DRP1 to FIS1 without affecting physiological mitochondrial fission. They have shown that the increase in the inflammatory response of astrocytes is in part mediated by fragmented mitochondria derived from microglia; these findings were mostly demonstrated using *in vitro* culture models. Their study also demonstrated that activated astrocytes exhibit several fragmented mitochondria as well; however, the potential role of fragmented mitochondria released from astrocytes in inflammatory cascades has not been investigated. Future studies are required to better understand the crosstalk between microglia and astrocytes through fragmented mitochondria in neuroinflammation.

The development of status epilepticus causes alterations in mitochondrial functions compromising astrocytic viability. An animal study conducted by Ko et al. (2016) showed that widespread reactive astrogliosis with reduced length of astrocytic mitochondria is observed in the dentate gyrus, whereas elongated mitochondria are found in autophagic astrocytes in the cornu ammonis 1 (CA1) region of hippocampus under the status epilepticus condition. The region-specific alterations in mitochondrial dynamics in astrocytes correlate with DRP1 phosphorylation, which is regulated by mitochondrial cyclin-dependent kinase 5 (CDK5) that promotes mitochondrial fission. However, pharmacological inhibition of CDK5 (Olomoucine and roscovitine) and mitochondrial fission (Mdivi-1) was found to ameliorate astrogliosis and cellular apoptosis following status epilepticus (Ko et al., 2016; Hyun et al., 2017).

Chronic neuroinflammation is one of the hallmarks of PD pathophysiology (Lee et al., 2019). Clinical reports and animal studies have indicated that gliosis and an increase in the levels of inflammatory mediators are common features of PD pathogenesis (Wang et al., 2015). The persistent release of proinflammatory molecules, including cytokines and chemokines, by activated microglia and reactive astrocytes, results in the enhancement of dopaminergic neuronal degeneration in the substantia nigra. Focusing on astrocytes, pro-inflammatory astrogliosis has been observed in the brain of patients with PD, suggesting that reactive astrocytes are involved in the alteration of immune processes in PD pathophysiology (Yamada et al., 1992). It has been reported that an excess amount of misfolded α-synuclein in the brain of PD mouse model triggers astrocytes to alter into inflammatory phenotypes inducing widespread astrogliosis, microglial activation, increased expression levels of TNF-α and IL-6, and subsequent degeneration of dopaminergic neurons (Gu et al., 2010; Fellner et al., 2013). Another report indicated that the phosphatase and tensin homolog-induced putative kinase 1 (PINK1)/parkin pathway regulates mitochondrial dynamics and function of mammalian cells (Yu et al., 2011). Consistent with this, loss of function in PINK1/parkin due to mutations has been reported to induce mitochondrial damage and suggested to be involved in the early onset of PD (Exner et al., 2012). Remarkably, parkin dysfunction in astrocytes has been reported to impair mitochondrial function, contributing to the pathogenesis of PD (Ledesma et al., 2002). This finding suggests that parkin dysfunction might be involved in defective mitophagy in astrocytes and subsequent PD pathogenesis. Future study is required to investigate the role of mitophagy in astrocytes in neurodegenerative diseases. It has also been reported that parkin is involved in the astrocytic inflammatory response. In this regard, astrocytic activation by IL-1β induced a decrease in the level of parkin (Khasnavis and Pahan, 2014). Furthermore, another PD-related gene *DJ-1* has been demonstrated to alter mitochondrial dynamics in cultured astrocytes, resulting in a lower neuroprotective ability (Lev et al., 2013). Similarly, a decreased level of DRP1 is also found in the astrocytes of patients with PD (Hoekstra et al., 2015), implying an impairment of mitochondrial dynamics in the brain astrocytes of these patients. However, the mechanistic correlation between astrocytic mitochondrial dynamics and their inflammatory activation in PD pathophysiology remains largely unknown. Future studies are necessary to identify the mitochondrial pathways that regulate astrocytic phenotypes associated with neuroinflammation in PD.

### Mitochondrial Bioenergetic Perturbation During Inflammatory Activation of Astrocytes

Multiple lines of evidence suggest that glial (microglia and astrocytes) phenotypic transition is governed by a mitochondrial metabolic shift particularly from oxidative phosphorylation to glycolysis (Jiang and Cadenas, 2014; Nair et al., 2019). Unlike microglia, the astrocytes are primarily glycolytic, demonstrating a lower oxidative metabolic rate (Walz and Mukerji, 1988). The phenotypic alterations observed in reactive astrocytes can be associated with mitochondrial metabolism; however, further studies are required to elucidate the role of mitochondrial bioenergetic distress in the inflammatory activation of astrocytes under different conditions.

#### Triggers and Mediators of Mitochondrial Dynamics and Metabolic Changes in Inflammatory Astrocytes

Astrocytes undergo a gradual activation process in response to diverse stimuli, including injury, stroke, inflammation, neurotoxins, pathogens, over-nutrition, genetic factors, and β-amyloid peptides (Afridi et al., 2020). Natural byproducts, including ROS such as hydroxyl radicals (OH^−^), superoxides (O^2−^), and reactive nitrogen species such as Nitric oxide (NO) play vital roles in cell signaling, gene transcription, and microbial defense (Rizor et al., 2019). Several lines of evidence indicated the role of NO in regulating DRP1 activity and mitochondrial fission (Barsoum et al., 2006; Bossy et al., 2010). A study by Motori et al. (2013) has revealed that inflammation-induced NO production is required for DRP1 activation and subsequent mitochondrial fragmentation in astrocytes. These fragmented mitochondria in astrocytes exhibit increased ROS generation and compromised ATP production, which lead to the activation of the NF-κB pathway and the release of proinflammatory cytokines, initiating a toxic feedforward loop of chronic neuroinflammation toward neurotoxicity (Motori et al., 2013; Kaur et al., 2019). Furthermore, studies have reported that NO-induced activation of hypoxia-inducible factor 1-alpha and 5′ AMP-activated protein kinase signaling pathways enhance the expression of glycolytic genes in reactive astrocytes (Almeida et al., 2004; Brix et al., 2012). However, further research is required to correlate mitochondrial dysfunction and involvement of other inflammatory signaling pathways such as mitogen-activated protein kinase, c-Jun N-terminal kinases, and extracellular-signal-regulated kinase (Park et al., 2013, 2015) in reactive astrocytes.

Mitochondria are dynamic cellular organelles, and the balance between fusion and fission is firmly connected with their bioenergetics. Accumulating studies have linked mitochondrial dynamics to the balance between energy demand and nutrient supply, suggesting the changes in mitochondrial structure as a mechanism for bioenergetic adaptation to metabolic demands (Liesa and Shirihai, 2013). However, alterations in both mitochondrial dynamics and bioenergetic metabolism have been correlated and considered as prominent events in the pathogenesis of neuroinflammation and neurodegenerative diseases. In the context of inflammatory astrocytes, alterations in both mitochondrial dynamics and bioenergetics have been observed in several studies (Motori et al., 2013). However, further investigation is required to understand the molecular pathways underlying the coordination between mitochondrial dynamics and the energy metabolism in health, which are essential for the characterization of astrocytic inflammatory activation in metabolic and neurodegenerative diseases. In the light of limited knowledge, it has been suggested that the possible regulation of mitochondrial fusion by the energy state is brought about by the changes in the mitochondrial membrane potential-dependent cleavage of OPA1 (Ishihara et al., 2006). Moreover, fission can also be controlled by energy-dependent processes through the cAMP-dependent activation of DRP1 (Duvezin-Caubet et al., 2007). Proteomic analyses have demonstrated that posttranslational modifications of DRP1, OPA1, and MFN2 may interfere with the regulation of mitochondrial dynamics and the subsequent oxidative phosphorylation capacity in response to cellular energy state and nutrient availability (Benard et al., 2007). These findings may help future studies identify the molecular link between mitochondrial bioenergetics and dynamics in astrocyte function in health and diseases.

### Conclusions and Future Perspective

**Figure 1:**
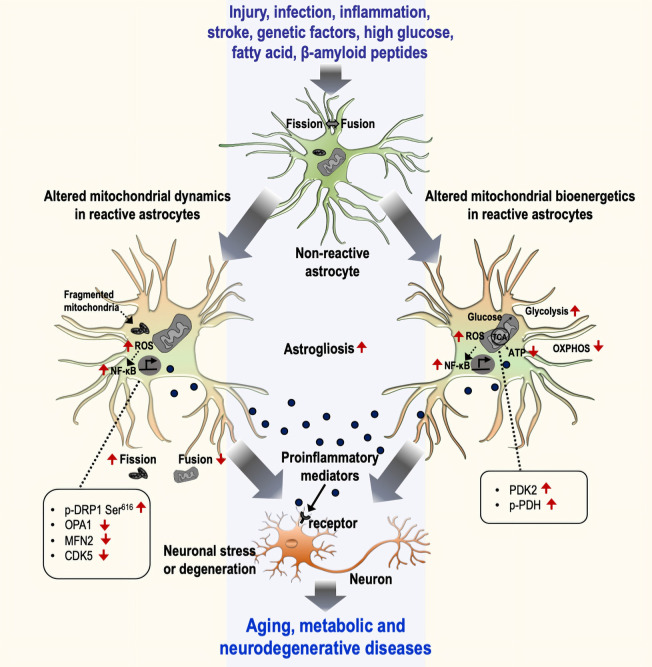
Mitochondrial dynamics and bioenergetics in inflammatory astrocytes and neurological disorders. Astrocytes undergo a gradual activation process in response to various stimuli, including injury, infection, inflammation, stroke, genetic factors, high glucose, fatty acid, and β-amyloid peptides, that leads to the induction of inflammatory phenotypes through the alteration of mitochondrial dynamics (left) and bioenergetics (right). The alteration of mitochondrial dynamics is represented by an increase in fission and a decrease in fusion events characterized by changes in related proteins [p-DRP Ser^616^, optic atrophy 1 (OPA1), MFN2, and CDK5], culminating in the increased number of fragmented mitochondria. Similarly, altered mitochondrial bioenergetics is represented by increased glycolysis and decreased mitochondrial oxidative phosphorylation. The change in the bioenergetic process is mediated by the regulation of mitochondrial proteins such as the upregulation of PDK2 and p-PDH. This phenomenon induces astrocytes toward their inflammatory activation through the excessive production of reactive oxygen species (ROS) and the subsequent activation of the NF-κB pathway. Reactive astrocytes release diverse pro-inflammatory mediators and induce inflammatory neuronal stress or toxicity associated with aging and metabolic and neurodegenerative diseases. OXPHOS, oxidative phosphorylation.

The role of altered mitochondrial dynamics and bioenergetics in diverse neuropathologies is being excitedly investigated. Alteration of mitochondrial dynamics and bioenergetics in astrocytes has been implicated in neurometabolic and neurodegenerative conditions, including diabetes or obesity, aging, AD, HD, and PD (Table 1). Accumulating evidence suggests that dysregulation of mitochondrial fission and fusion events, defective mitophagy, and impaired bioenergetics are largely associated with the inflammatory activation of astrocytes. Consistent with this, altered mitochondrial morphology and function cause cellular stress through the activation of diverse signaling pathways, suggesting that mitochondrial dysfunction is associated with the changes in astrocytic inflammatory phenotypes (Figure 1). Although no selective drug or treatment strategies are targeting specifically astrocytic mitochondria at present, current and future studies may increase the feasibility of astrocyte-based therapeutic strategies targeting mitochondrial pathways to prevent and alleviate astrocyte-mediated neuroinflammation and subsequent neuropathologies.

**Table 1:** Experimental findings related to mitochondrial dynamics and bioenergetics in inflammatory astrocytes and neuropathologies.

Disease/ neuropathologies	Model/stimuli	Mitochondrial dynamics and metabolic changes	Suggested mechanism	Remarks	References
Brain injury	Stab wound injury *in vivo*. LPS + IFN-γ stimulation *in vitro*.	Excessive mitochondrial fission, enhanced glycolysis, and reduced mitochondrial respiration.	Increased levels of p-DRP1 (Ser^616^), mitochondrial ROS, and iNOS.	Impaired mitophagy and mitochondrial motility, multiple forms of astrocyte reactivity in different brain areas.	Motori et al. (2013)
Alzheimer disease	5XFAD mice and cellular models. _0_Aβ_42_ or LPS or nigericin stimulation *in vitro*.	Pathological mitochondrial fragmentation, lower ATP levels, loss of normal inner mitochondrial membrane polarization, and increased mitochondrial ROS production.	DRP1-FIS1-mediated mitochondrial fission.	Astrocyte activation to A1 proinflammatory state and a release of proinflammatory cytokines such as TNF-α and IL-1β.	Joshi et al. (2019)
Huntington disease	R6/2 mice and cellular models. LPS or nigericin stimulation *in vitro*.	Pathological mitochondrial fragmentation, lower ATP levels, loss of normal inner mitochondrial membrane polarization, and increased mitochondrial ROS production.	DRP1-FIS1-mediated mitochondrial fission.	Astrocyte activation to A1 proinflammatory state and a release of proinflammatory cytokines such as TNF-α and IL-1β.	Joshi et al. (2019)
Amyotrophic lateral sclerosis	SOD1-G93A mice and cellular models. LPS or nigericin stimulation *in vitro*.	Pathological mitochondrial fragmentation, lower ATP levels, loss of normal inner mitochondrial membrane polarization, and increased mitochondrial ROS production.	DRP1-FIS1-mediated mitochondrial fission.	Astrocyte activation to A1 proinflammatory state and a release of proinflammatory cytokines such as TNF-α and IL-1β.	Joshi et al. (2019)
Parkinsonian syndrome	Chronic manganese-induced neurotoxicity.	Increased mitochondrial circularity and fragmentation, decrease in basal mitochondrial oxygen consumption, and reduced level of ATP.	Decreased level of MFN2.	Inflammatory activation of astrocytes characterized by an elevated GFAP expression and increased expression of proinflammatory factors such as TNF-α, IL-1β, IL-6, IL-12, and NOS2.	Sarkar et al. (2018)
Status epilepticus (SE)	Pilocarpine-induced SE in rat.	Increased mitochondrial fission and reduced fusion along with the reduced length of astrocytic mitochondria.	Increased expression of p-DRP1 (Ser^616^) by mitochondrial cyclin-dependent kinase 5 and decreased levels of p-DRP1 (Ser^637^) and OPA1.	Reactive astrogliosis.	Ko et al. (2016) and Hyun et al. (2017)
Aging	Aged rats (6–18 months old). IL-1β or TNF-α stimulation *in vitro*.	Higher mitochondrial respiration rate.	Age-dependent increase in H_2_O_2_ generation and NOX expression.	Functionality switch of astrocytes to a reactive state.	Jiang and Cadenas (2014)
Diabetes and obesity	Mouse models of STZ and HFD-induced diabetes and obesity. High glucose or palmitate stimulation *in vitro*.	Altered glycolytic metabolism is characterized by an increased level of lactate and extracellular acidification rate.	The metabolic shift from oxidative phosphorylation to glycolysis by upregulation of PDK2 and p-PDH (S^293^ and S^300^).	Increased levels of cytokines (TNF-α, IL-1β, and IL-6) and gliosis.	Rahman et al. (2020)

A study conducted by Jiang and Cadenas (2014) demonstrated a metabolic–inflammatory axis in primary astrocytes. These cells exhibit increased responses with age to inflammatory cytokines such as IL-1β and TNF-α. Furthermore, exposure of astrocytes to IL-1β and TNF-α alters their mitochondrial bioenergetics, suggesting that the alterations in mitochondrial aerobic metabolism and inflammatory responses are a mutual process and support the functionality switch of astrocytes to a reactive state with age. This metabolic change is associated with an age-dependent increase in hydrogen peroxide generation and activation of NF-κB signaling. A previous study involving a seahorse analysis of bioenergetic status in manganese-treated astrocytes demonstrated an impaired basal mitochondrial oxygen consumption and adenosine triphosphate (ATP)-linked respiration rates, which have been correlated with manganese-induced astrocytic inflammatory phenotypes characterized by increased expression of proinflammatory factors such as TNF-α, IL-1β, IL-6, IL-12, and NOS2 (Sarkar et al., 2018). Also, a recent study showed that metabolic reprogramming of astrocytes governs hypothalamic inflammation and its sequelae in diabetes. In that study, Rahman et al. (2020) identified that pyruvate dehydrogenase (PDH) kinase (PDK)-2, a key regulator of the mitochondrial gatekeeping enzyme PDH, induces a metabolic shift from oxidative phosphorylation to glycolysis in astrocytes that contributes to neuroinflammatory responses characterized by increased levels of inflammatory cytokines (TNF-α, IL-1β, and IL-6) and gliosis; this further modulated the neuropeptidergic circuitry in the hypothalamus associated with altered feeding behavior in diabetes. However, astrocyte-specific genetic ablation and pharmacological inhibition of PDK2 by AZD7545 and lactate dehydrogenase (a glycolytic enzyme) by oxamate or GSK2837808A were found to ameliorate the diabetes-induced hypothalamic inflammation and the subsequent metabolic syndrome, suggesting that PDK2 in hypothalamic astrocytes can be a potential therapeutic target for neuroinflammation and associated metabolic disorders.

## Author Contributions

MR conducted the literature review, formulated, and wrote the manuscript. KS edited the manuscript and was involved in all aspects of manuscript preparation. All authors contributed to the article and approved the submitted version.

## Conflict of Interest

The authors declare that the research was conducted in the absence of any commercial or financial relationships that could be construed as a potential conflict of interest.
